# Thematic series – Low back pain

**DOI:** 10.1186/s13013-016-0108-5

**Published:** 2017-01-18

**Authors:** Dino Samartzis, Theodoros B. Grivas

**Affiliations:** 1 Department of Orthopaedics and Traumatology, The University of Hong Kong, Pokfulam, Hong Kong, SAR China; 2 Orthopaedics and Trauma Department, “Tzaneio” General Hospital of Piraeus, Piraeus, 18536 Greece

**Keywords:** Spine, Low back pain, Risk factors, Prevention, Diagnosis, Treatment, Outcome, Disc Degeneration

Low back pain is the world’s most disabling condition, involving all populations and crossing all boundaries [1, 2]. Such pain leads to tremendous socioeconomic and health-care consequences. In the United States alone, the direct and indirect costs associated with low back pain surpass 90 billion USD per year, with similar adjusted rates in different countries [3]. Low back pain is the second condition following the common cold that motivates individuals to seek medical consultation, and is the top debilitating condition in individuals over the age of 45 [4–7]. Such a condition can lead to diminished quality of life, decreased activity, loss of wages, and psychological distress [4–7]. In fact, chronic low back pain may even age the brain faster and destroy critical brain tissue in comparison to asymptomatic individuals [8].

Throughout the years, various conservative and non-conservative measures have been developed to treat low back pain [5]. However, the outcome of such treatment is often not predictable or satisfactory [6]. Furthermore, the use of opioid consumption to manage low back pain has escalated in recent years, prompting concern of its use and abuse [9, 10]. As such, identifying risk factors that assist in preventing low back pain as well as predicting outcomes from various management options has taken center-stage. Large-scale epidemiological and patient cohorts (i.e. “big data” platforms) have been developed that aim to have a better understanding of imaging, clinical and “omics” profiles towards understanding risk/prediction and susceptibility to low back pain and its severity [11–20]. Novel treatments are being pursued worldwide to target potential pain generating sources in hopes to treat such pain and improve patient outcomes and quality of life. Animal models of pain have been and are currently being developed to address the pathogenesis of pain, understand pain pathways and mechanisms, and facilitate therapeutic drug discovery and testing [21, 22]. The role of abnormal biomechanics, occupational, lifestyle and psychosocial exposures have been and continue to be addressed to enhance understanding of the causes and consequences of low back pain [11, 12, 17, 18, 23–30]. In short, the implications of low back pain to patients as well as to clinicians and researchers alike are substantial, and will continue to be of importance.

Due to the gravity that low back pain imposes upon society, and its implications upon clinical management, health-care costs, and various research platforms, the *Scoliosis and Spinal Disorders* journal has initiated a “Low Back Pain” thematic series. The journal will welcome all types of articles and research designs, with the exception of case reports, that focus on low back pain. We believe this series will broaden the understanding of low back pain, and provide new insights into prevention, refined diagnoses, management options, novel therapeutics and outcomes.
